# An efficient and accurate 2D human pose estimation method using VTTransPose network

**DOI:** 10.1038/s41598-024-58175-8

**Published:** 2024-03-31

**Authors:** Rui Li, Qi Li, Shiqiang Yang, Xin Zeng, An Yan

**Affiliations:** 1 School of Mechanical and Precision Instrument Engineering, Xi’an University of Technology, Xian, 710000 China; 2 Xi’an People’s Hospital, Xi’an, China

**Keywords:** Human pose estimation, Transformer, Twin attention, Feature fusion, Computer science, Information technology, Scientific data

## Abstract

Human pose estimation is a crucial area of study in computer vision. Transformer-based pose estimation algorithms have gained popularity for their excellent performance and relatively compact parameterization. However, these algorithms often face challenges including high computational demands and insensitivity to local details. To address these problems, the Twin attention module was introduced in TransPose to improve model efficiency and reduce resource consumption. Additionally, to address issues related to insufficient joint feature representation and poor network recognition performance, the enhanced TransPose model, named VTTransPose, replaced the basic block in the third subnet with the intra-level feature fusion module V block. The performance of the proposed VTTransPose model was validated on the public datasets COCO val2017 and COCO test-dev2017. The experimental results on COCO val2017 and COCO test-dev2017 indicate that the AP evaluation index scores of the VTTransPose network proposed are 76.5 and 73.6 respectively, marking improvements of 0.4 and 0.2 over the original TransPose network. Additionally, VTTransPose exhibited a reduction of 4.8G FLOPs, 2M parameters, and approximately 40% lower memory usage during training compared to the original TransPose model. All the experimental results demonstrate that the proposed VTTransPose is more accurate, efficient, and lightweight compared to the original TransPose model.

## Introduction

Human pose estimation is the study of algorithms or systems for recovering joint and torso poses based on observed data from images, which has led to one of the very challenging and significant research directions in the field of computer vision because of the large variety of different joint scales in the human body and the interference of the scene for joint points in the real environment^1^. The human pose estimation algorithms can be classified into two main categories: 2D pose estimation algorithms and 3D pose estimation algorithms according to the number of dimensions that represent the human pose features. Among them, 2D pose estimation algorithms mainly obtain the human key point coordinates and skeletal correspondence by obtaining the position information of human key points on 2D images and the position and orientation information of human limb trunks, and the accuracy of this correspondence directly affects the results of human pose estimation^2^.

Traditional two-dimensional pose estimation methods mostly use probabilistic graphical models or image structure models, but the results are not satisfactory, and in recent years, many scholars have started to apply deep learning ideas to models for pose estimation^3^.

Deep learning-based two-dimensional human pose estimation algorithms use convolutional neural networks (CNNs) to simulate the human visual system by superimposing multiple convolutional layers to obtain rich features under different receptive fields. CNNs are widely used in pose estimation tasks due to their advantages of capturing the human features required for pose estimation and high recognition accuracy^4–6^. Intending to solve the body occlusion problem, Chen et al. constructed the Cascaded Pyramid Network (CPN)^7^ network in 2019, CPN includes two stages: GlobalNet and RefineNet. GlobalNet fuses high and low-level features to correlate contextual information and locate simple human body articulation points, and RefineNet combines features from all levels processed by convolution to deal with occluded articulation points. To alleviate the impact of different scales of different human joint points, in 2019, Sun et al. proposed the HRNet algorithm^8,9^, which fuses semantic and positional information of images at different resolutions by parallel branching, and finally outputs a high-resolution feature map. The model improves the aggregation ability of low-level positional features and high-level semantic features by fusing features of different scales.

The above CNN-based methods are characterized by the ability to learn features at different scales through cascade structure, which facilitates the model to learn discriminative information on different semantic spaces. However, it is also because such methods need to continuously superimpose the network depth to gradually increase the receptive field and obtain the global feature information, which leads to the network becoming bulkier and heavier. To alleviate these problems, researchers have focused on the transformer architecture in the field of natural language processing. The core concept of the Transformer model lies in its reliance on attention mechanisms to entirely capture dependency relationships within input sequences, thereby eschewing the use of recurrent neural networks (RNNs) or convolutional neural networks (CNNs). Transformer-based models achieve this by requiring only a single self-attention layer to learn associations between any pair of features, thus enabling the modeling of relationships from local features to global context with a reduced parameter count. Moreover, it learns more about the interconnections between different features, not only relying on the data itself, which has better generalizability. Inspired by the success of transformer structures in other vision tasks^10–13^, a variety of different visual transformer structures have been successfully used for pose estimation. Based on a cascaded regression mechanism, Li and his colleagues^14^ proposed a PRTR-based pose estimation network that uses an encoder-decoder structure to progressively predict human keypoints. To enhance the feature representation capability of the network for the highly fine-grained task of pose estimation, Yuan et al.^15^ proposed an HRFormer to adapt the pose estimation task by introducing high-resolution representation into the visual transformer through a multi-resolution parallel transformer module. Different from the pure Transformer architecture, both TokenPose and TransPose human pose estimation models proposed by Li et al.^16^ from Tsinghua University and the group led by Yang from Southeast University^17^ respectively, utilize visual transformers to refine features extracted by CNN, thereby complementing the two network architectures. Ye et al. proposed the DistilPose model, which bridges the gap between heatmap-based and regression-based methods, transferring models based on heatmaps to models based on regression, effectively balancing speed and accuracy^18^. Cheng et al. introduced GTPose, which integrates Transformer and graph convolutional networks, establishing a topological relationship model between keypoints, learning feature representations, and achieving precise keypoint localization^19^. Among these, the TransPose human pose estimation network model organically combines CNN's excellent handling of local features with the Transformer's excellent modeling capability for global features. Introducing the Transformer into human pose estimation not only excellently accomplishes the pose estimation task but also reveals how the self-attention mechanism captures the global relationship between various joints of the human body. This CNN-transformer hybrid architecture provides a new solution approach for pose estimation tasks, demonstrating excellent performance both in pose estimation effectiveness on datasets like MS COCO^20^ and MPII^21^, as well as in interpretability.

Nevertheless, insufficiencies still exist in this approach. First, the extensive feature maps in the pose estimation task and the inherent nature of the self-attention computing mechanism will impose heavy computational costs and consume large computational resources. Second, the scale of human joints varies greatly, and this CNN-transformer architecture is not sufficient to make a delicate local representation of joints at various scales. To address the above problems, this paper proposes an improved pose estimation algorithm called VTTransPose based on TransPose.

The contribution of this work can be summarized as follows.

Firstly, to alleviate the problem of large consumption of time and computational resources in human pose estimation tasks due to the extensive feature map, and the inherent characteristics of the self-attention mechanism. In this paper, we propose to introduce twin attention from SOTR^22^ into TransPose and replace the self-attention mechanism in the original encoder layer to significantly reduce memory consumption and thus improve network efficiency. In addition, in order to enhance the special extraction capability without introducing more operations. In this paper, two 3 × 3 depthwise separable convolutions connected by leaky ReLU^23^ are introduced after the twin attention of each transformer encoder layer as a useful complement to the attention mechanism to enhance the representation of joint features.

Secondly, to address the issue of scale differences in the original network, which hindered the precise localization of keypoints for body joints with significant scale variations, this paper was inspired by RSN^24^ and designed a local feature enhancement module called the V block. It features a parallel multi-branch structure to fuse features with the same spatial size, thereby obtaining refined local features and enhancing the network's ability to localize keypoints.

The pose estimation effect of VTTranspose was tested on the COCO data set, and the AP (Average precision) of COCO val2017 and COCO test-dev2017 was 76.5AP and 73.6AP, respectively. Compared with the original Transpose network improved by 0.4 and 0.2. In addition, compared to the original network, VTTranspose's floating point operations (FLOPs) are reduced by 4.8G, the number of parameters is reduced by 2M, and the video memory occupancy during training is reduced by about 40%. Compared with other SOTA methods, VTTranspose proposed in this paper has a competitive performance.

The following paper is organized as follows: In “Method”, the Method part will first give a brief description of the overall structure of VTTranspose. Later, the two improved modules of Twin attention, improved transformer encoder layer, and Backbone after the introduction of the V block will be explained in detail. In “Experimental and result analysis”, the experimental and result analysis section will validate the VTTransPose algorithm developed in this paper on the COCO dataset and compare it with other excellent pose estimation algorithms. In “Conclusion”, the conclusion section will summarize the algorithm of this paper and give an outlook on future research.

## Method

### VTTransPose network structure

**Figure 1:**
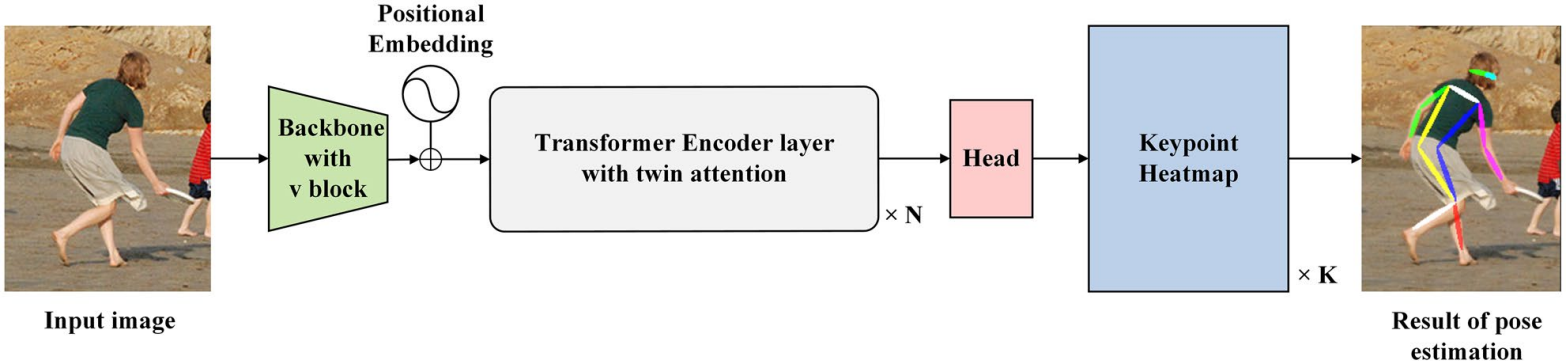
VTTransPose network structure diagram.

The VTTransPose network architecture proposed in this paper is an improvement upon the TransPose model^17^. TransPose is a detection model that introduces Transformer principles into human pose estimation. The model mainly consists of three parts: a backbone network for extracting mid-level human pose features from input images, a transformer encoder for modeling global relationships among the joint features output by the backbone network, and a head for predicting the positions of human keypoints. The structure of VTTranspose is illustrated in Fig. 1.

Take a human body pose image $I\in {R}^{3\times {H}_{I}\times {W}_{I}}$ as an example. Feed it into the VTTranspose network. After Backbone processing, the low and middle-level feature maps of human joints based on this image are output, whose mathematical characterization method is ${X}_{f}\in {R}^{d\times H\times W}$, and the channel dimension has been changed to d through the 1 × 1 convolution. Then, the image feature map is flattened into a sequence $X\in {R}^{L\times d}$, where L is equal to H × W. After that, it is fed into N Transformer encoder layers for processing. Finally, the output $E\in {R}^{L\times d}$ is fed into a head to predict Kkeypoint heatmaps $P\in {R}^{K\times {H}^{*}\times {W}^{*}}$, where the default settings are $H*$, $W*=HI/4$, WI/4. After obtaining the heatmap of the different joints, the position with the highest thermal value in the heatmap is selected to obtain the joints' coordinates.

### Transformer encoder layer after twin attention is introduced

The self-attention mechanism, as the core of the transformer, has received a lot of attention for its excellent modeling ability for the connection between long-range features^24^. Along with the excellent global modeling ability of self-attention, there is also a large consumption of computational resources due to its complex matrix operations. Therefore, to alleviate this problem, this paper introduces the twin attention mechanism into transformer encoders to replace the original self-attention computational mechanism and make the network more efficient and resource-friendly.

#### Self-attention mechanism

**Figure 2:**
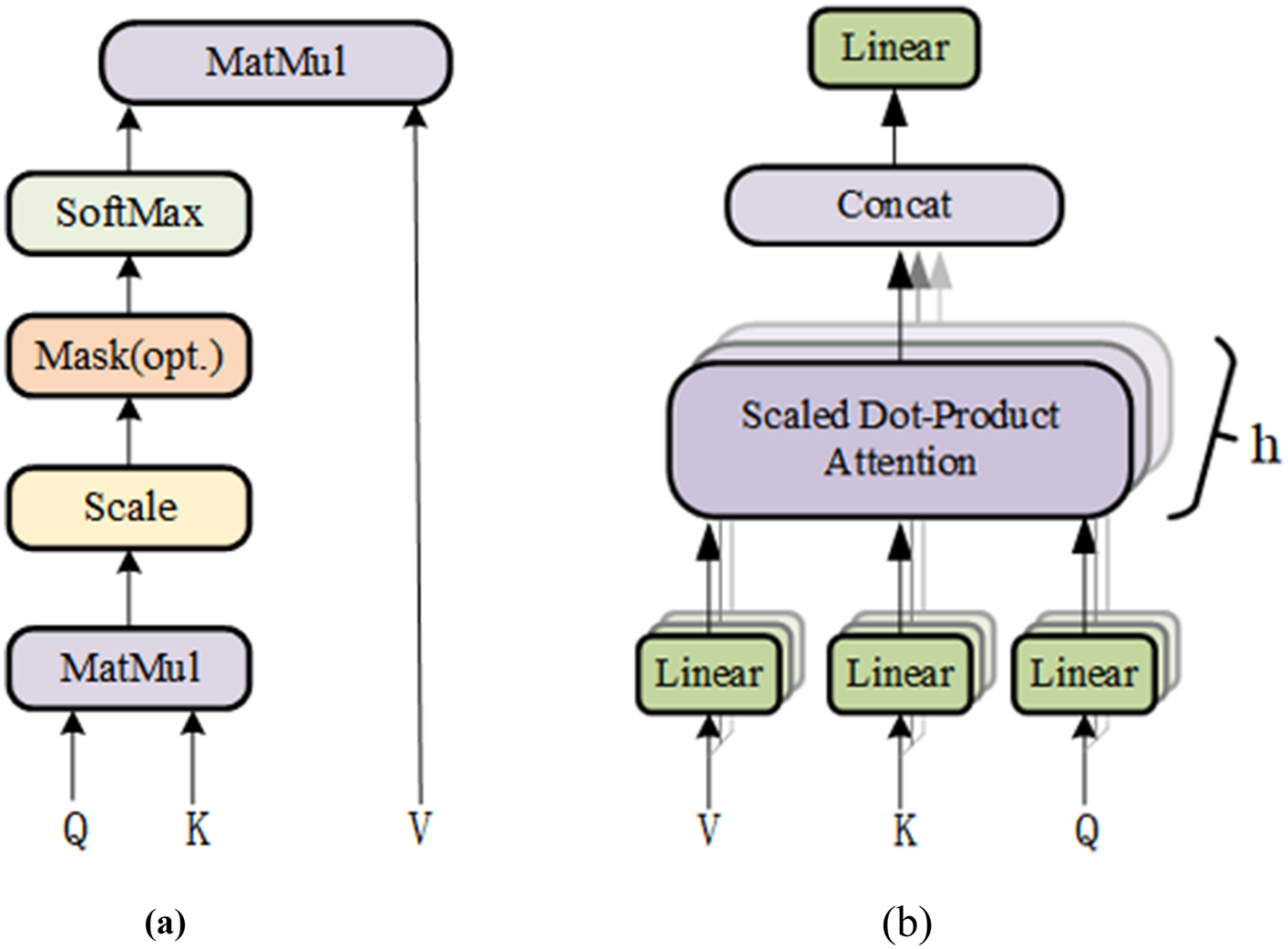
The attention calculation model. (**a**) Scaled dot-product attention, (**b**) Multi-head attention.

The self-attention mechanism, as the core of the transformer, can be further divided into single-head self-attention and multi-head self-attention according to the number of attention heads^22^. The calculation process of the single-head self-attention mechanism is as follows: first, the input sequence $X\in {R}^{L\times d}$ is multiplied by the three weight matrices $Wq, Wk, Wv\in {R}^{d\times d}$, then we can get the queries, keys, values matrices $Q, K, V\in {R}^{L\times d}$ to calculate the attention value, the schematic diagram of self-attention is depicted in Fig. 2a.

Firstly, the correlation score matrix $A\in {R}^{L\times L}$ between each input feature ${x}_{i}\in {R}^{d}$ in ${\text{X}}$ is calculated using the dot product of vectors. This is, every vector ${q}_{i}\in {R}^{d}$ in Q is calculated with every vector ${k}_{i}\in {R}^{d}$ in $K$. Specifically in matrix form, it can be expressed in Eq. (1).

1 $$\mathbf{A}=\mathbf{Q}{\mathbf{K}}^{\mathbf{\top }}$$

After that, all correlation scores ${w}_{i}\in {R}^{L}$ in the result matrix A are normalized to enable the gradient stable during training, which can be expressed by Eq. (2).

2 $$\mathbf{A}=\mathbf{A}/\sqrt{{d}_{k}}$$

where, dk denotes the dimension of k, whose value is d in this paper.

Finally, each ${w}_{i}$ is converted to a probability distribution between [0, 1] by the softmax function and then multiplied by the corresponding ${{\text{v}}}_{{\text{i}}}\in {\mathbb{R}}^{{\text{d}}}$, the attention value can be obtained, and the overall calculation process can be expressed by Eq. (3).

3 $$\mathbf{Z}={\text{Attention}}(\mathbf{Q},\mathbf{K},\mathbf{V})={\text{softmax}}(\frac{\mathbf{Q}{\mathbf{K}}^{\boldsymbol{\top }}}{\sqrt{{d}_{k}}})\mathbf{V}$$

where $Z\in {R}^{L\times d}$ is the attention matrix.

The multi-head self-attention mechanism, involves processing the original input sequence through multiple sets of self-attention operations. Subsequently, the results of each set of self-attention are concatenated and subjected to a linear transformation to obtain the final output. As illustrated in Fig. 2b, it utilizes h sets of $Wq$, $Wk$, $Wv$ to derive multiple sets of $Q$,$K$, $V$. Then, according to Eq. (3), the attention matrix is computed for each set separately. Finally, the obtained multiple matrices are concatenated^24^. Through these computations, the relationships between any two input vectors can be obtained, which helps overcome the issue of the model excessively focusing on its own position when encoding information at the current position.

#### Twin attention mechanism

**Figure 3:**
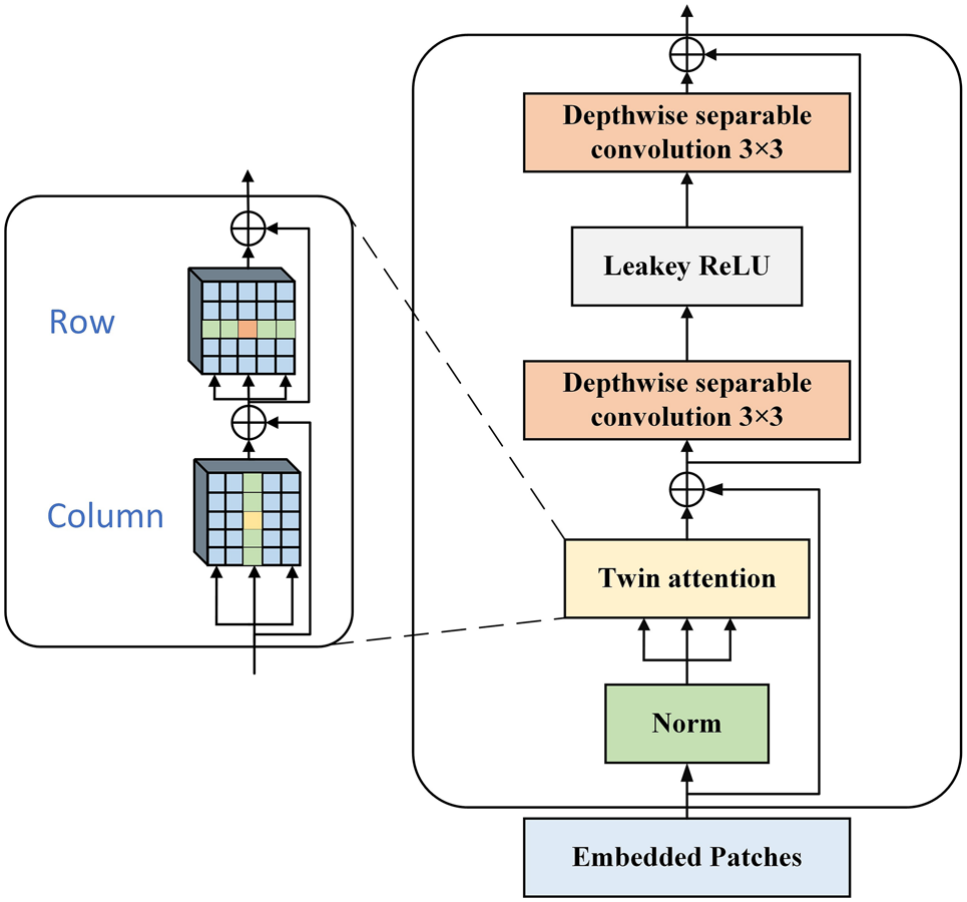
Transformer encoder with twin attention and depthwise separable convolution.

As shown in the left side of Fig. 3, Twin attention is similar to convolution decomposition in CNN, the design idea of twin attention is to decompose the original self-attention into two steps, and then obtain a sparse representation of the original attention matrix. It first computes the attention of each column in the input matrix, while keeping the elements in different columns non-interfering with each other, and this strategy can aggregate the contextual information among the elements on the horizontal scale. After computing the attention in the column direction, a similar computation strategy is executed for each row along the row direction to obtain the attention of each row to establish a perfect connection between the features at the vertical scale. Connecting the attention in these two scales sequentially, a sparse representation equivalent to self-attention is obtained, which has a global receptive field with information in both horizontal and vertical dimensions and can model the global connections of the input features.

For the input two-dimensional human posture feature map ${X}_{f}\in {R}^{d\times H\times W}$, it will be decomposed into N*N patches ${{\text{P}}}_{{\text{f}}}\in {\mathbb{R}}^{{\text{d}}\times {\text{N}}\times {\text{N}}}$, and then stack them in the vertical and horizontal directions respectively. To preserve the position information of the features, position embedding is added to the blocks stacked along the two directions, and the position embedding space in the column and row directions are ${\text{d}}\times 1\times {\text{N}}$ and ${\text{d}}\times {\text{N}}\times 1$. Through this strategy, Twin attention can effectively reduce the memory consumption and computational complexity from the standard self-attention $\mathcal{O}({({\text{H}}\times {\text{W}})}^{2})$ to $\mathcal{O}({{\text{H}}\times {\text{W}}}^{2}+{{\text{W}}\times {\text{H}}}^{2})$.

Assuming that the width and height of the input two-dimensional human pose features are 48, 64, the reduction in memory consumption and computational complexity of twin attention reaches 96% compared with the standard self-attention mechanism, which can certainly enhance the efficiency of the network greatly.

#### Depthwise separable convolution blocks

**Figure 4:**
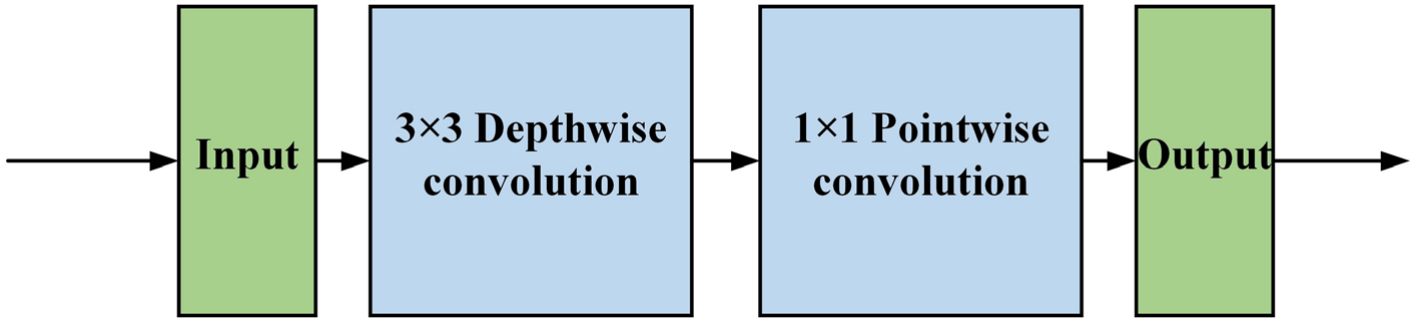
Diagram of depthwise separable convolution.

Considering that the sparse attentional representation may lead to the degradation of network detection performance, this paper uses two depthwise separable convolution blocks connected by leaky ReLU to replace the FFN in the original transformer encoder to enhance the feature representation capability of the network. The structure of the depthwise separable convolution block is shown in Fig. 4, which divides the traditional convolution operation into two steps. First is depthwise convolution, where one convolution kernel is responsible for only one channel, so the number of convolution kernels needed is the same as the number of input channels. After that, the generated feature maps are sent to pointwise convolution to aggregate the information on different channels. With this strategy, the depthwise separable convolution reduces the computational complexity from $O (K\times K\times {C}_{in}\times {C}_{out})$ to $O(K\times K\times {C}_{in}+{C}_{in}\times {C}_{out})$, so that it can provide a useful complement to the twin attention mechanism without introducing a high computational cost. where K represents the convolution kernel size, Cin represents the number of input feature channels, and Cout represents the number of output feature channels.

### TransPose backbone after the introduction of V block

After the introduction of twin attention, the transformer encoder layer is significantly less resource-intensive and the efficiency of the network can be improved. However, the backbone network is not sufficient for the extraction of low and medium-level features at multiple scales. Although the HRNet as Backbone fuses features with different resolutions to obtain spatial feature information with different receptive fields through the parallel multi-subnet structure, the transition of receptive fields between different subnets is not smooth enough and the interval of receptive fields is large. For human joints with different scales, such as the nose with a smaller scale and the chest with a larger scale, the network needs a larger range and more scales of receptive fields to extract feature information at different scales^7^. Reference^25^ suggests that merging features within layers with the same resolution can achieve more refined local feature representations, preserving more accurate spatial feature information, which is more conducive to precise localization of keypoints. Therefore, this paper proposes a layer-wise multi-branch feature fusion module called the V block to refine the network's receptive field spacing, increase the overall receptive field range of the network, and obtain fine representations of local features.

#### Backbone structure

There are two series of TransPose network backbones: ResNet-S and HRNet-S. In order to keep the high-resolution representation of the feature, HRNet-S is chosen for the study in this paper. As shown in the upper part of Fig. 4, HRNet-S mainly consists of one stem unit, three stages, and two transition units. The network can be divided into three sub-networks according to the feature map resolution, and the feature map resolutions of sub-networks 1,2,3 are gradually reduced to 1/4, 1/8, 1/16 of the input images, respectively.

The input image will be downsampled to 1/4 of the original image in the spatial size and increased in the channel dimension through the stem unit and the first stage, and then a parallel sub-network is added by doubling downsampling in each transition part. Except for the first stage which is composed of bottleneck blocks, the second and third stages are composed of one and four high-resolution modules respectively, where each high-resolution module consists of four basic blocks. Whenever the input features pass through a high-resolution module, a feature fusion operation between different subnets is performed immediately afterward. It is this design of constantly fusing different resolution features while maintaining high-resolution feature representation that allows HRNet to perform well in various vision tasks. Note that at the end of the third stage, only the feature map of the first subnet is output, which has the highest resolution and fully fuses the features of the three different resolution subnets.

#### Structure of the intra-level feature fusion module V block

**Figure 5:**
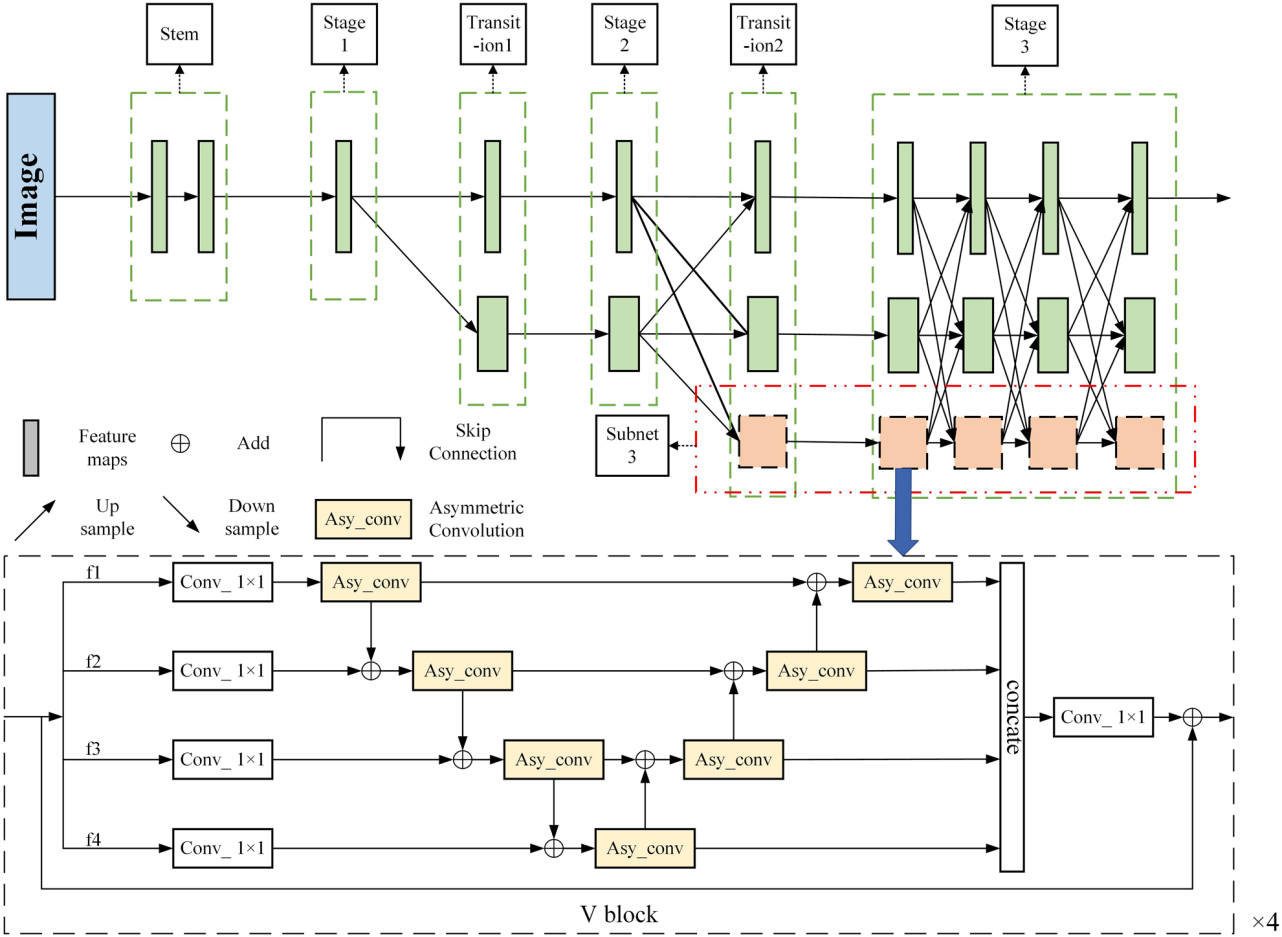
The HRNet-S framework after V block is introduced.

The proposed V block structure in this paper is shown in the black dashed box at the bottom of Fig. 5. For the third subnet of low-resolution human pose input features ${{\text{F}}}_{{\text{in}}}$, V block first divides them into 4 branches ${f}_{i}(i=1, 2, 3, 4)$ in the channel dimension, Then, the features of each of the four branches are fed into a 1 × 1convolution for processing. After that, the output of each branch is processed by a $3\times 3$ asymmetric convolution in turn (the asymmetric convolution structure is explained below). The output feature ${f}_{i}{\prime}(i=1, 2, 3, 4)$ is added to the next branch, inspired by Bi-FPN^26^, in this paper, after the forward summation is completed, a set of reverse sequential summation operations are performed, and each summation operation is followed by a $3 \times 3$ asymmetric convolution block to process the summed features. Output features ${y}_{i}(i=1, 2, 3, 4)$ will be fed into a $1\times 1$ convolution after concatenated, and an identity connection is employed as the HRNet Basic block. The V block is named because the arrangement of the asymmetric convolutional blocks is similar to the V in the alphabet. The V block can be expressed by the following formula:

4 $${F}_{out}={F}_{in}+{K}_{\mathrm{1,1}}\odot ({\sum }_{i=2}^{4}({K}_{ac}\odot ({y}_{i}+{K}_{ac}\odot ({f}_{i-2}{\prime}+{K}_{\mathrm{1,1}}\odot ({f}_{i-1}))))+{y}_{4})$$

where ${{\text{F}}}_{{\text{out}}}$ represents the output feature map of a single V block, ${{\text{F}}}_{{\text{in}}}$ represents the input feature map, $\sum \text{()}$ represents the concation operation, and ${{\text{f}}}_{{\text{i}}}^{\mathrm{^{\prime}}}$ is the feature of each branch after the first asymmetric convolution block processing, when $i=2$, let ${f}_{i-2}{\prime}=0$, ${{\text{K}}}_{\mathrm{1,1}}$ represents the convolution operation using a convolution kernel of 1 × 1, and $\odot$ represents the convolution operation, and ${{\text{K}}}_{{\text{ac}}}$ represents the asymmetric convolution operation using a set of convolution kernels of $(3\times 1, 1\times 3)$*.*

**Figure 6:**
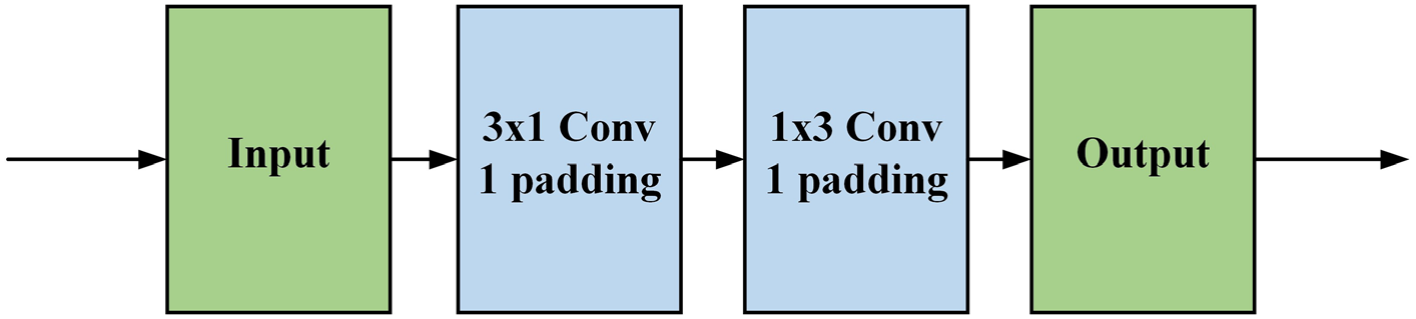
Asymmetric convolution.

To reduce the network's parameters and fully explore its feature extraction capabilities, this paper utilizes asymmetric convolutions in the V block instead of standard convolutions. Asymmetric convolutions decompose the standard $d\times d$ convolution into $1\times d$ and $d\times 1$ convolutions, reducing the parameter count. By performing convolution operations of varying degrees in different directions and positions, asymmetric convolutions can better capture subtle features and structural information in the input data, thereby enhancing the model's understanding of the input and improving the network's perception of the local range of each keypoint. The structure is shown in Fig. 6, for the input feature ${f}_{input}$, it will first undergo a convolution with a convolution kernel size of 3 × 1 for processing, and then send the processed features into a convolution kernel with a convolution kernel size of 1 × 3 for further feature extraction. Through this strategy, the same feature extraction effect as standard convolution can be achieved. And the number of parameters can be reduced. The asymmetric convolution processing flow can be expressed by the following formula:

5 $${f}_{ac}={K}_{\mathrm{1,3}}\odot ({K}_{\mathrm{3,1}}\odot {f}_{input})$$

where $\odot$ denotes the convolution, ${{\text{f}}}_{{\text{input}}}$ denotes the input feature map, ${{\text{f}}}_{{\text{ac}}}$ denotes the output feature map, and ${{\text{K}}}_{{\text{i}},{\text{j}}}$ denotes the convolution operation with the size of the kernel $i\times j$.

#### The receptive field analysis

In the literature^7^, it is pointed out that a larger range, as well as more scales of receptive fields, are essential to extract feature information at different scales, so in this paper, the analysis of different modules of receptive fields is carried out in this section. First, the receptive field calculation can be expressed by the following equation.

6 $${l}_{k} = {l}_{k-1} + [({f}_{k} - 1)*{\prod }_{i=1}^{k-1}{s}_{i}]$$

where ${{\text{l}}}_{{\text{k}}}$ represents the receptive field of the ${{\text{k}}}$ th layer, ${{\text{f}}}_{{\text{k}}}$ denotes the kernel size of the ${k}$ th layer, ${{\text{s}}}_{{\text{i}}}$ denotes the stride of the ${i}$ th layer. Since the complete structure of each network is complex and inconvenient for complete analysis and comparison, only the relative receptive fields in a single block are considered and compared in this paper. Every ${f}_{k}$ is 3 and ${{\text{s}}}_{{\text{i}}}$ is 1. Thus, Eq. (6) can be simplified to Eq. (7)

7 $${l}_{k} = {l}_{k-1} + 2$$

The relative receptive fields of V block and the two blocks in HRNet can be calculated by using Eq. (7), as shown in Table 1, which shows that the proposed V block has a larger receptive field compared with the two blocks in HRNet, which is beneficial for the network to learn more semantic discriminative information and thus obtain more accurate joint localization and classification.

**Table 1:** The receptive field comparison between V block and the block in HRNet.

Architecture	y1	y2	y3	y4
HRNet_Bottleneck block	3	3	3	3
HRNet_Basic block	3, 5	3, 5	3, 5	3, 5
V block	3, 15	5, 13	7, 11	9

## Experiment and result analysis

The COCO 2017 dataset is selected to train and test the improved model in this paper, and the effectiveness of this paper's model is demonstrated by comparing it with other excellent models.

### COCO data set and evaluation index

The COCO 2017 dataset contains more than 200,000 images and 250,000 human instances, each labeled with the location of 17 keypoints. This dataset is widely used for tasks such as target detection, human pose estimation, and semantic segmentation. The COCO train2017 dataset is used to train the proposed model in this paper, which includes 57K images and 150K human instances. The model of this paper is evaluated on the val2017 set and the test-dev2017 set, where the val2017 set contains 5000 images and the test-dev2017 set contains 20K images. This paper uses AP (average precision), AR (average recall), Params(parameter), and FLOPs (floating point operations) to evaluate networks.

Assessment is based on object keypoint similarity (OKS) in the node detection task of the COCO dataset. OKS represents how close the predicted key points are to the actual situation, and a higher OKS score means a higher overlap between the predicted key points and the actual situation, that is, the prediction is more accurate. The calculation method is shown in Eq. (8).

8 $$OKS=\frac{{\sum }_{i}^{N}{\text{exp}}\left(-\frac{{d}_{i}^{2}}{2{S}^{2}{\sigma }_{i}^{2}}\right)\delta \left({v}_{i}>0\right)}{\sum_{i}^{N}\delta \left({v}_{i}>0\right)}$$

where ${d}_{i}$ is the Euclidean distance between the i th prediction key point and the generalized truth value; $S$ represents the scaling factor, $\sigma$ represents the normalization factor of the ${\text{i}}$ key point; ${{\text{v}}}_{{\text{i}}}$ is the visibility parameter of the ith key point. ${{\text{v}}}_{{\text{i}}}$ is 0 when the key point does not exist, 1 when the key point exists but is blocked, and 2 when the key point exists and is visible. The function of $\delta$ is to select the key point of existence, which has two values. When ${{\text{v}}}_{{\text{i}}}$>0, the value is 1, otherwise it is 0; ${\text{N}}$ represents the number of key points, which in COCO is 17.

This paper uses the standard average precision (AP) value based on OKS as an evaluation indicator. AP can be used to evaluate the network's ability to detect key points. The higher the value, the better the detection performance. AP is calculated as shown in Eq. (9).

9 $$\begin{array}{c}{{\text{AP}}}^{{\text{T}}}=\frac{{\sum }_{i}^{M}{\delta }_{i}(OKS>T)}{M}\end{array}$$

where, $T$ is the threshold set when calculating AP; ${\delta }_{i}$ is used to determine the relationship between the *OKS* score of the $i{\prime}{\text{th}}$ individual and the size of threshold $T$. If $OKS>T$ is met, ${\delta }_{i}$ is set to 1, otherwise it is set to 0. $M$ is the number of human instances in the test set.

### Training details

This paper follows a top-down paradigm for human pose estimation. The training samples are single human images after cropping. All input images will be resized to resolution. In this paper, we use the same data expansion, human detection results, and coordinate decoding strategy as in^17^. In this paper, the Adam optimizer is used to train the model with a training period of 240 epochs, the batch size is set to 16. The cosine annealing learning rate decay is used to change the learning rate from 0.0001 to 0.00001. The environment configuration for network training is shown in Table 2.

**Table 2:** Experimental training environment.

Environment configuration	
System	Windows10
GPU	P106-100
Memory size	6 GB
CPU	Intel(R) Core(TM) i5-4460 CPU @ 3.20 GHz
Python	3.7
Torch	1.2.0
CUDA	10.0

### VTTranspose pose estimation effect test

**Figure 7:**
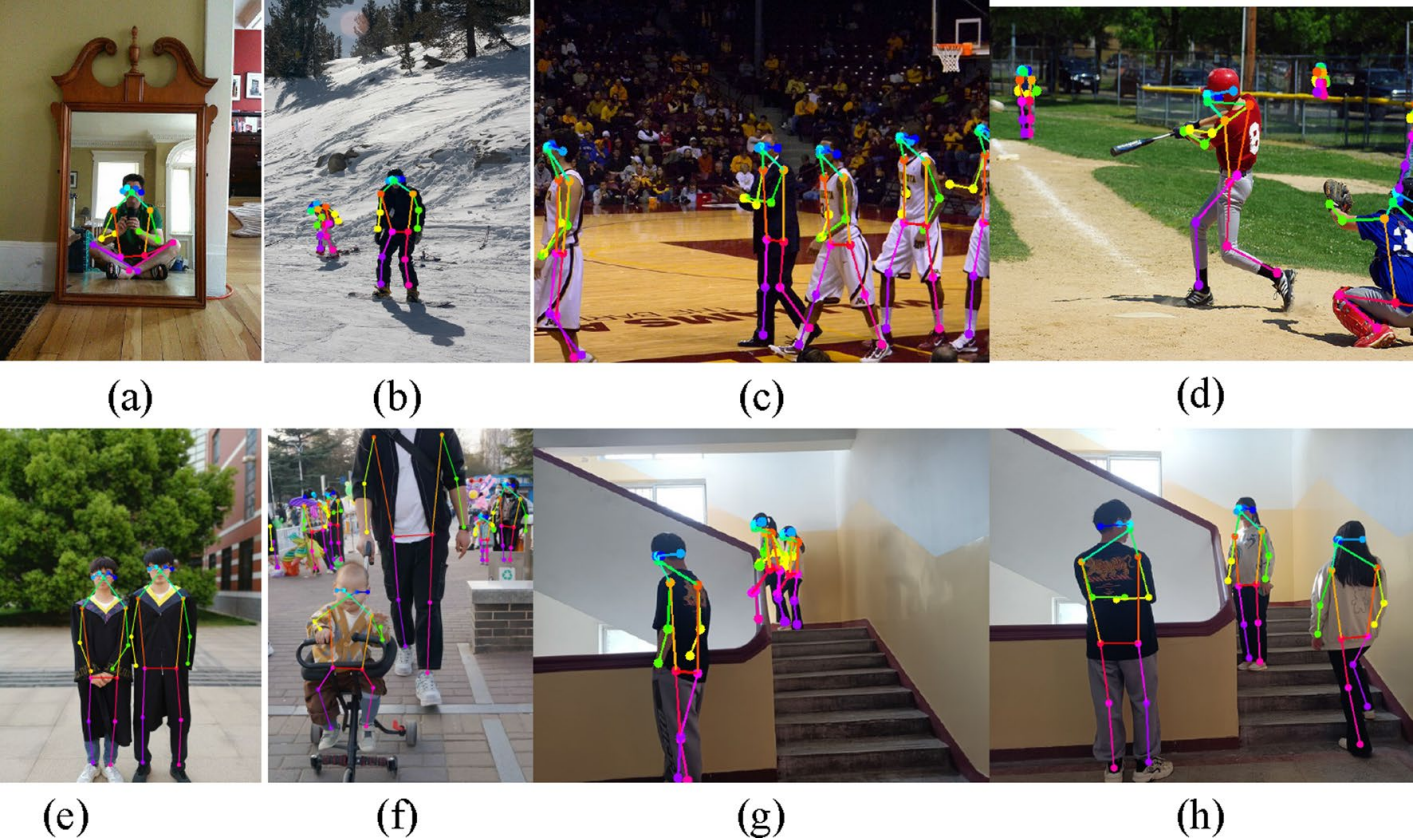
VTTransPose pose estimation effect demonstration.

Figure 6 shows the display image of the human pose estimation results of VTTransPose. Figure 7a–d show the pose estimation results on four images selected in COCO val2017, and Fig. 7e–h show the pose estimation results on the images taken in the actual scene. It can be observed that VTTransPose has good pose estimation results in different human scales, different human postures, and with slight occlusion, but the results are not good when dealing with large occlusion and human overlap, for example, the pose estimation results of two farther overlapping human bodies at the corner of the stairs in Fig. 7g.

### Validation results on the COCO val2017 and the COCO test-dev2017 datasets

In this paper, VTTranspose's ability to complete the node detection task is tested respectively on two data sets of COCO val2017 and COCO test-DEV2017, and the test results are shown in Tables 3 and 4 respectively.

**Table 3:** Comparisons on the COCO validation set.

Method	Input size	AP	AR	#Params (M)	FLOPs (G)
SimpleBaseline-Res50^27^	256 × 192	70.4	76.3	34.0	8.9
SimpleBaseline-Res101^27^	256 × 192	71.4	76.3	53.0	12.4
SimpleBaseline-Res152^27^	256 × 192	72.0	77.8	68.6	35.3
TransPose-R-A3*^17^	256 × 192	71.5	76.9	5.0	5.4
TransPose-R-A3^17^	256 × 192	71.7	77.1	5.2	8.0
TransPose-R-A4^17^	256 × 192	72.6	78.0	6.0	8.9
HRNet-W32^8^	256 × 192	74.4	79.8	28.5	7.2
HRNet-W48^8^	256 × 192	75.1	80.4	63.6	14.6
TokenPose-B^16^	256 × 192	74.7	80.0	13.5	5.7
DistilPose-S^18^	256 × 192	71.6	–	5.4	2.38
DistilPose-L^18^	256 × 192	74.4	–	21.3	10.33
GTPose-B^19^	256 × 192	75.0	80.1	13.5	–
TransPose-H-A4^17^	256 × 192	75.3	80.3	17.3	17.5
TransPose-H-A6^17^	256 × 192	75.8	80.8	17.5	21.8
TransPose-H–S^17^	256 × 192	74.2	78.0	8.0	10.2
VTTranspose (ours)	256 × 192	74.6	78.5	6.0	5.4

**Table 4:** Comparisons on the COCO test-dev set.

Method	Input size	#Params (M)	FLOPs (G)	AP	AP_0.5_	AP_0.75_	AP_M_	AP_L_
G-RMI^28^	353 × 257	42.6	57	64.9	85.5	71.3	62.3	70.0
Integral^29^	256 × 256	45.0	11.0	67.8	88.2	74.8	63.9	74.0
CPN^7^	384 × 288	58.8	29.2	72.1	91.4	80.0	68.7	77.2
RMPE^30^	320 × 256	28.1	26.7	72.3	89.2	79.1	68.0	78.6
SimpleBaseline^27^	384 × 288	68.6	35.6	73.7	91.9	81.1	70.3	80.0
HRNet-W32^8^	384 × 288	28.5	16.0	74.9	92.5	82.8	71.3	80.9
HRNet-W48^8^	256 × 192	63.6	14.6	74.2	92.4	82.4	70.9	79.7
HRNet-W48^8^	384 × 288	63.6	32.9	75.5	92.5	83.3	71.9	81.5
DarkPose^31^	384 × 288	63.6	32.9	76.2	92.5	83.6	72.5	82.4
TokenPose^16^	256 × 192	13.5	5.7	74.0	91.9	81.5	70.6	79.8
DistilPose-S^18^	256 × 192	5.4	2.38	71.0	91.0	78.9	67.5	76.8
DistilPose-L^18^	256 × 192	21.3	10.33	73.7	91.6	81.1	70.2	79.6
GTPose-B^19^	256 × 192	13.5	–	74.5	92.2	82.2	70.7	79.8
TransPose-H-A4^17^	256 × 192	17.3	17.5	74.7	91.9	82.2	71.4	80.7
TransPose-H-A6^17^	256 × 192	17.5	21.8	75.0	92.2	82.3	71.3	81.1
TransPose-H–S^17^	256 × 192	8.0	10.2	73.4	91.6	81.1	70.1	79.3
VTTranspose (ours)	256 × 192	6.0	5.4	73.6	91.4	81.1	70.1	79.6

Compared to transpose-H–S. Compared to models such as HRNet-W48, TokenPose, GTPose, TransPose-H-A4, and TransPose-H-A6, although VTTransPose may have slightly lower AP, its parameter count and computational complexity are only 1/10 to 1/2 of these models. Compared to other models in the table, VTTransPose achieves higher accuracy with lower parameter counts and computational complexity.

On the COCO test-dev2017 dataset, all models are evaluated based on the object detection results obtained from the same human object detector achieving 60.9 AP. As shown in Table 4, among models with the same input size, the proposed VTTranspose achieves competitive performance with 73.6 AP while having the fewest parameters and the lowest computational complexity.

### Ablations

In order to verify the respective effects of two improvement modules in VTTransPose, the following experiments were carried out on COCO val2017. Firstly, only introduce the improved transformer encoder layer to the original TransPose, then train the model and test its network performance. On this basis, V block is introduced to Backbone and network performance is detected after training. The results are shown in Table 5.

**Table 5:** Ablation study on the two improvement modules.

Model	Backbone	Params (Mb)	Memory (batch size = 4) (Mb)	AP (coco val gt bbox)
TransPose-H–S	HRNet-S-W32	8	3503	76.1
TransPose-H–S + twin attention	HRNet-S-W32	8	1953	76.3
VTTransPose	HRNet-S-W32 + V block	6	2007	76.5

As can be seen from Table 5, firstly, after twin attention is introduced based on the original TransPose model, the memory occupied during the model training decreases significantly, with a decrease ratio of 44.2%, which greatly improves the training efficiency; and due to the addition of depthwise separable convolution, the feature extraction ability of the model is enhanced, and the AP index is improved compared with the original model by 0.2. After that, the AP index is improved by 0.2 with the introduction of V block, which slightly increases the memory consumption, but reduces the number of model parameters by 25% with the introduction of asymmetric convolution, so that the model achieves a balance of accuracy and the number of parameters.

## Conclusion

This paper presents a top-down human pose estimation model VTTransPose. First, to reduce the computational complexity of self-attention in the transformer, save computational resources, and speed up the training and convergence process, the self-attention in the original TransPose network is replaced by the twin attention computing mechanism, which can reduce the memory and computational complexity from O((H*W)2) to O(H*W2 + W*H2). And the depthwise separable convolution is added after twin attention to replace the MLP module to enhance the local feature capture capability with a very small amount of computation. Later, to enhance the feature extraction ability and expression ability of the model for the fine-grained task of keypoint detection, the intra-level feature fusion module V block was introduced into the third subnet of HRNet-S in TransPose to achieve intra-level feature and inter-level feature fusion of the network. In addition, to enhance the feature extraction capability of the network while reducing the number of parameters, the standard convolution within the V block is replaced with an asymmetric convolution, and the number of parameters is reduced by such convolutional decomposition without reducing the accuracy. The validation results on COCO val2017 and COCO test-dev2017 datasets show that VTTransPose has lower memory consumption, higher training efficiency, and higher accuracy compared with the original model. The proposed model also has a competitive performance when compared with other good models.

Although the comprehensive performance of the model proposed in this paper is good, the limitation to the inherent CNN-Transformer fusion framework leads us to make only small improvements in various aspects and does not reach SOTA. Therefore, in the future, we will make a study on how to better fuse CNN and transformer architectures in pose estimation tasks.

## Data Availability

The data presented in this study are available on request from the corresponding author. The data are not publicly available due to [In order to adapt to our study, we processed the dataset].
